# Synthesis and Properties of Polyimide Silica Nanocomposite Film with High Transparent and Radiation Resistance

**DOI:** 10.3390/nano11030562

**Published:** 2021-02-24

**Authors:** Jindong Huang, Guanglu Zhang, Beiping Dong, Juncheng Liu

**Affiliations:** 1 School of Materials Science and Engineering, Tiangong University, Tianjin 300387, China; jdhuang@tiangong.edu.cn (J.H.); zgltg@tiangong.edu.cn (G.Z.); 1931025097@tiangong.edu.cn (B.D.); 2 School of Physical Science and Technology, Tiangong University, Tianjin 300387, China

**Keywords:** polyimide, nano-SiO_2_, in situ polymerization, transmittance, radiation resistance, mechanical properties

## Abstract

In order to prepare flexible glass cover sheet materials suitable for space solar cells, fluorinated diamine 2,2’-bistrifluoromethyl benzidine (TFDB) and fluorinated dianhydride 4,4’ (hexafluoroisopropyl) diphthalic dianhydride (6FDA) as the monomer, polyimide (PI)/SiO_2_ composite film was synthesized by in situ polymerization, and the influence of coupling agent and SiO_2_ nanoparticle content on the film structure and properties was studied. The results show that PI synthesized from fluorine-containing monomers has better light transmittance, and the highest transmittance can reach 91.4%. The average visible light transmittance of the composite film decreases with the increase of SiO_2_ content, and the transmittance of the film decreases less in the high-wavelength region and greatly decreases in the low-wavelength region. The tensile strength and elastic modulus of PI/SiO_2_ composite film increase with the increase of SiO_2_ content, first increase and then decrease, reaching the maximum when the content is 10%; while the elongation at break of the composite film gradually increases with the increase of SiO_2_ content reduce. The thermal stability of PI/SiO_2_ composite film increases with the increase of SiO_2_ content. The doping of nano-SiO_2_ significantly suppresses the influence of irradiation on the mechanical properties of the film.

## 1. Introduction

With the rapid development of aerospace technology, flexible space solar cells are urgently needed. Flexible solar cells need to be protected by glass cover sheets due to the presence of atomic oxygen (AO), ultraviolet rays, various cosmic ray radiation, and micrometeoroids in the space environment. The glass cover sheet of the flexible solar cell should possess sufficient flexibility and good mechanical properties, moreover, high light transmittance, resistance to ultraviolet rays and various cosmic rays. Therefore, it is urgent to find an alternative material for space solar cell glass cover sheets with the above comprehensive performances.

Polyimide (“PI” for short) is a type of polymer whose main chain is an aromatic ring and an imide ring as the main structural units. It has excellent comprehensive properties, such as high creep resistance, high electrical insulation, low thermal expansion coefficient, low dielectric constant, etc., with excellent high and low-temperature resistance and corrosion resistance. PI plays an increasingly important role in the fields of microelectronics, optical devices, power and electrical, aerospace and other fields [1,2,3]. However, besides flexibility, pure PI is difficult to meet other requirements of space solar cell glass cover sheets, such as high light transmittance and high radiation resistance.

In order to improve the light transmittance of PI, Tao et al. [4,5] prepared a series of polyimide hybrid films by polymerizing 2,2’-bis trifluoromethyl benzidine and 4,4’-hexafluoroisopropyl diphthalic anhydride and adding different contents of dodecyl triphenyl phospho-mica. The light transmittance of these hybrid films was up to 90% at 450 nm. The above polyimide membranes with fluorinated substituents introduced into the molecular chain generally have high transparency, excellent thermal stability and low dielectric constant. However, the relatively high synthesis cost of fluorinated monomers has limited the industrialization process of fluorinated polyimide membranes to some extent. In addition, the mechanical properties and thermal stability of the general fluorinated PI are relatively poor. Appropriate reinforcing materials should be introduced to improve the comprehensive properties of the composites.

In order to improve the radiation resistance of PI, Wang et al. [6] reported nano-SiO_2_ filled glass fiber/PI composite material, which was used as a resin for spacecraft. The research results displayed that the corrosion resistance of the composite material in the atomic oxygen environment was significantly enhanced, indicating that SiO_2_ was beneficial to enhance the system’s resistance to space radiation and atomic oxygen. Li et al. [7] prepared transparent PI film through the reaction between phosphorous-containing fluorine-containing aromatic diamine monomer 2,5-bis [(4-amino-2-trifluoromethylphenoxy)phenyl] diphenylphosphine oxide and the commercialized aromatic dianhydride. The obtained PI film had good solubility, heat resistance and mechanical properties in organic solvents but also exhibited that the introduction of diphenylphosphine oxide side groups (DPO), fluorine, all meta-position could improve the light transmittance and atomic oxygen resistance of the material.

In order to improve the mechanical properties of PI, Hu et al. [8] added carbon nanotubes to the polyimide and searched for the best ratio. Finally, the composite material with the best room temperature mechanical properties was obtained when 0.2% carbon nanotubes were added. Huang et al. [9] synthesized a new type of aminopropyl isobutyl POSS with single apex activity, which was used to form a composite film material with PMDA and ODA. This material showed excellent mechanical properties, with a tensile strength of 148 MPa and elongation at break up to 98%. Cheng Jingdong [10] used silica sol as the silicon source to prepare PI/SiO_2_ nano-hybrid films via in situ polymerization. With the increase of SiO_2_ content, the modulus of elasticity increased linearly. The tensile strength and elongation at break increased first and then decreased with the increase of SiO_2_ content and reached the maximum value when the SiO_2_ content was 3%. Li et al. [11] also found that the increase in the compatibility of SiO_2_ and PI increased the tensile strength and elongation at the break of the composite film. They reached the highest value when the content of SiO_2_ was 5% and decreased when the content continued to increase.

In order to improve the thermal stability of PI, Mazov et al. [12] used a phase inversion method to embed unprocessed single-walled carbon nanotubes as a filler into a polyimide matrix to synthesize a PI/r-MWCNTs film. It showed that with increasing the concentration of filler r-MWCNTs, the thermodynamic properties of PI/r-MWCNTs film would be improved. Shahram et al. [13] used pyromellitic dianhydride and common diamines as polymerized monomers to prepare nanoscale PI film, which illustrated that the film had good stability at a high temperature of 370 °C. Boroglu et al. [14] used ODA and PMDA as monomers, trimethoxyvinylsilane (TMVS) as an inorganic precursor to prepare PI/SiO_2_ hybrid films. The TGA test suggested that there was no weight loss before 400 °C, indicating the addition of SiO_2_ has improved the heat resistance of the film. With the increase of SiO_2_ content, the Tg of the film increased by 7~28 °C.

PI/SiO_2_ nanoparticle composite materials have become one of the research hotspots. The interface incompatibility between the inorganic filler SiO_2_ and the polyimide matrix results in the reduction of mechanical properties, polyimide molecular chain structure and crystallinity, and further affects the electrical insulation properties of polyimide materials. Because there are a large number of unsaturated residual bonds and hydroxyl groups in different bonding states on the surface of nano-SiO_2_, it is open to researchers to design the surface by controlling the balance of these unsaturated and unstable states to improve its compatibility with polymer polymerization. Through the design, the compatibility and dispersion of nano-SiO_2_ and the polymer matrix can be improved. The in situ polymerization process makes two different components to maximize the synergy effect. The in situ polymerization method is to add nanoparticles in the process of monomer polymerization and prepare nanocomposite materials through a further polymerization reaction. This method makes the nanoparticles more uniformly dispersed in the polymer matrix, which not only maintains the original structure and state of the nanoparticles but also enables the various groups on the surface of the nanoparticles to fully interact with the polymer molecules physically or chemically. Different interface operations between nanoparticles and polymer matrix have different effects on the properties of the matrix polymer.

In addition, the use of coupling agents is another dispersing method for chemical modification of the surface of nanoparticles after mechanical stirring and ultrasonic vibration. The surface free energy of the particles after surface modification will decrease, which benefits to reduce agglomerate. It is easy to obtain smaller nanoscale particles, which is conducive to the uniform dispersion of the nanoparticles in the organic matter and increases the interaction with the organic matrix. It is beneficial to obtain composite materials with better light transmittance and mechanical properties [15].

In this work, a series of PI/SiO_2_ composite films with different SiO_2_ content were prepared by in situ polymerization method, in which nano-SiO_2_ particles (about 15 nm in diameter) were used as the silicon source, and KH560 was selected as the silane coupling agent for surface modification, and 2,2’-bistrifluoromethylbenzidine (TFDB) and 4,4’ (Hexafluoroisopropyl) bisphthalic dianhydride (6FDA) was used as the monomer raw material. Fourier-transform infrared analysis (FT-IR), scanning electron microscopy (SEM), thermal weight loss analysis (TGA), ultraviolet-visible photometer and ultraviolet radiation simulator were used to investigate the bonding situation and microscopic morphology, thermal properties, optical properties, mechanical properties and UV resistance.

## 2. Materials and Methods

### 2.1. Preparation of PI/SiO_2_ Films

2,2’-Bistrifluoromethyl benzidine (TFDB, analytical purity 99%), 4,4’ (hexafluoroisopropyl) diphthalic acid Anhydride (6FDA, analytical purity 99%), and SiO_2_ nanoparticles(particle diameter of about 15 nm) were purchased from Shanghai Macklin Biochemical Technology Co., Ltd. (Shanghai, China). N, N’-Dimethylacetamide (DMAc, analytical purity 99%) and silane-coupling agent (KH-560, 98%) were purchased from Shanghai Jingchun Biochemical Technology Co., Ltd. (Shanghai, China). The molecular formula of the above chemical agents is shown in Figure S1. The XRD spectrum of the SiO_2_ nanoparticles we purchased is shown in Figure S2.

We weighed the nano-SiO_2_ particles of the required components in a drying beaker and dried it at 120 °C for 2 h. We weighed a certain mass of diamine in the drying beaker and dried it at 120 °C for 4 h. We weighed a certain mass of the dianhydride (molar ratio of TFDB and 6FDA = 1:1.02) and placed it in a drying beaker, and dried it at 180 °C for 4 h. The above raw materials were all dried under a normal laboratory atmosphere. The sample number and raw material ratio were shown in Table S1.

The dried SiO_2_ nanoparticles were dispersed in an appropriate amount DMAc at room temperature until a homogeneous solution was obtained. Then, a silane coupling agent (KH560) was added to the above solution with ultrasonic treatment for 2 h. After the SiO_2_ nanoparticles were uniformly dispersed and dissolved in DMAc, the dried diamine was added with continuously stirring. A colorless and transparent solution was obtained. During the whole process, the temperature was kept at 0 °C, and the atmosphere was nitrogen-protected. We added the dried dianhydride into the beaker in batches and added the next batch once it was completely dissolved. When approaching the equivalent point (molar ratio of TFDB and 6FDA = 1:1.02), the viscosity suddenly increased, and the phenomenon of Weissenberg pole-climbing occurred. For ease of laying the film, DMAc solvent could be appropriately added to keep the solid content of the system at about 10%. After this, the above solution was continuously stirred at room temperature for 24 h until a colorless or light yellow transparent solution formed, which was further allowed to stand at 0 °C for 4 h.

The 5 cm × 5 cm transparent glass plate was first cleaned by alcohol and dried in an oven at 80 °C for 30 min. A certain amount of polyamic acid (PAA)/SiO_2_ in DMAc solution was coated on the glass plate by using a glass rod. Then, we used an automatic film scraper to control the thickness of the film to about 20 μm. The above sample was placed in a 40 °C oven for 2 h. Then, then a stepped heating method (60 °C, 1 h; 70 °C, 1 h; 80 °C, 1 h; 100 °C, 1 h; 200 °C, 1 h; 300 °C, 1 h; 350 °C, 1 h) was used for the thermal imidization treatment. After the temperature of the sample decreased to room temperature, the sample was soaked in a distilled water bath for about 10 min. After the film was separated from the glass plate, the film was soaked in acetone solution for about 5 min, and then was dried in an oven at 80 °C for 10 min. The obtained PI/SiO_2_ composite film was stored in a sealed bag for testing. Moreover, the PI/SiO_2_ composite films with 5%, 10%, 20% and 30% SiO_2_ doping were labelled PIS05, PIS10, PIS20 and PIS30, respectively.

### 2.2. Characterization

The molecular structure, microstructure, thermal stability and optical transmittance performance of PI/SiO_2_ composite films were measured using a Fourier-transform infrared spectrometer (Nicolet iS50, Thermo Fisher, Waltham, MA, USA), scanning electron microscope (Gemini SEM500, ZEISS, Heidenheim, Germany), simultaneous thermal analysis (STA 449F5, NETZSCH, Bavaria, Germany), ultraviolet-visible-near-infrared photometer (UH4150, HITACHI, Tokyo, Japan) and UV Solutions, which is the supporting software of UH4150. The mechanical performance was measured on the material universal testing machine (AGS-X 50N, SHIMADZU, Tokyo, Japan). The PI/SiO_2_ films can be cut into 5 cm × 1 cm dumbbell-shaped splines. The tension was 10 mm/min. The radiation resistance of the PI/SiO_2_ films was tested using an ultraviolet lamp (HJ-1402, Cnlight, Foshan, China) to simulate a space irradiation experiment to examine changes in the light transmittance and mechanical properties of the composite film before and after irradiation.

## 3. Results and Discussion

**Figure 1:**
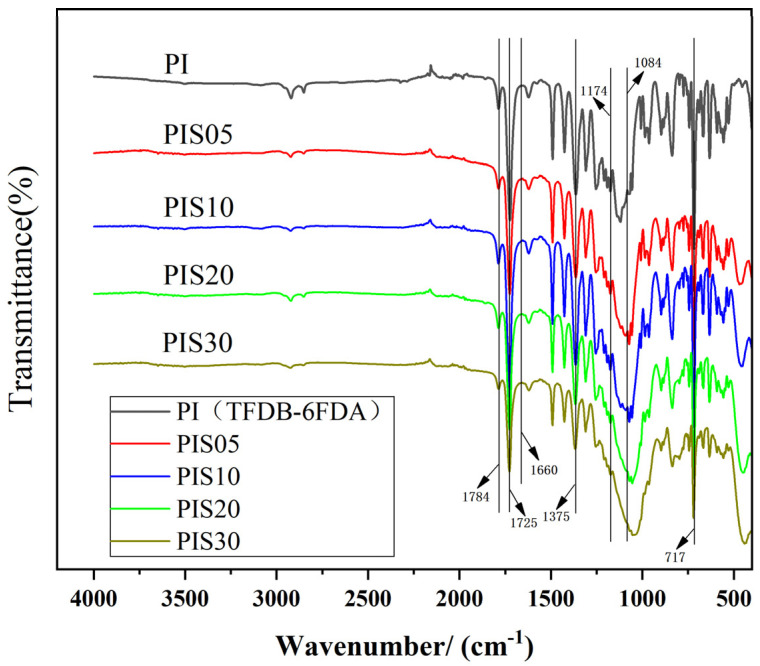
FT-IR spectra of polyimide (PI)/SiO_2_ films with various SiO_2_ contents.

The changes in the absorption peaks of the corresponding functional groups of PI and PI/SiO_2_ nanocomposite films were analyzed by using an FT-IR infrared spectrometer. Some of the most commonly used wave numbers for studying the imidization process are shown in Table S2. The FT-IR spectra of the composite films with different SiO_2_ content are shown in Figure 1. There are four main peaks for all the composite films with different SiO_2_ content located at 1784 cm^−1^, 1725 cm^−1^, 1375 cm^−1^ and 717 cm^−1^, corresponding to asymmetry extensional vibration absorption peak of C=O, the symmetrical extensional vibration absorption peak of C=O, the extensional vibration absorption peak of imine ring C–N–C, and the flexural vibration absorption peak of C=O. In addition, there is no absorption peak of amide bond at 1660 cm^−1^ in all samples, indicating that the thermal imidization process was complete [16].

There is a clear difference at 1000–1100 cm^−1^ between PI and PI/SiO_2_ composites films after comparing all the FT-IR spectra. The characteristic absorption peak of the Si–O–Si bond appears at 1084 cm^−1^. With the increase of SiO_2_ content, the characteristic absorption peak of the Si–O–Si bond gradually broadens. The asymmetric stretching vibration absorption peak of the cyclic Si–O–Si bond appears at 1174 cm^−1^, suggesting that the organic/inorganic components in the composite system exist in the form of chemical bonds, forming a crosslinked spatial network structure, namely PI/SiO_2_ strong interaction composite structure.

**Figure 2:**
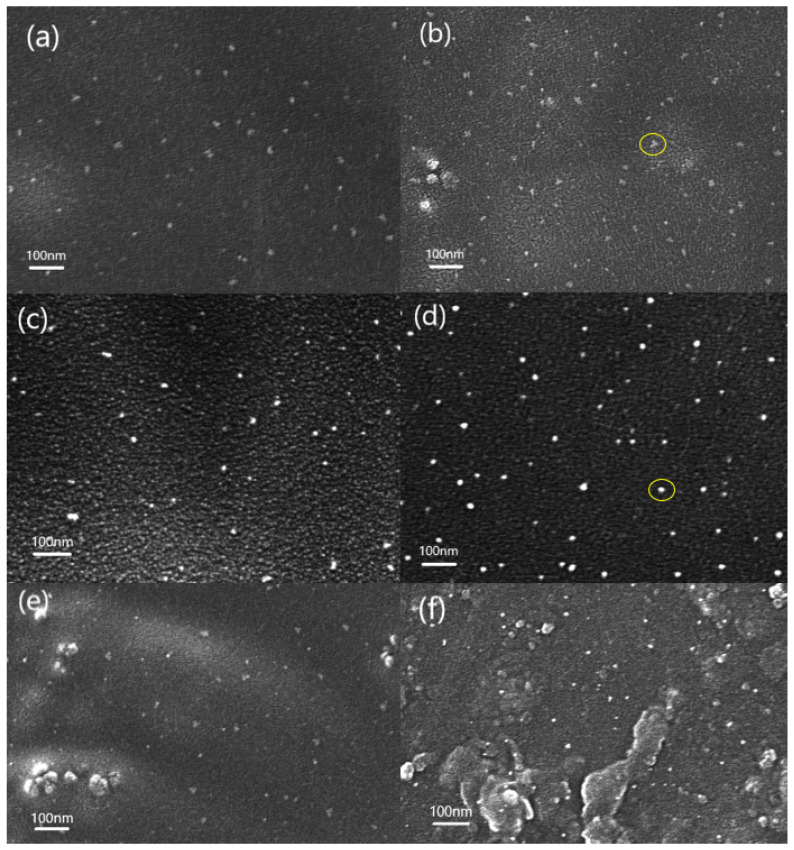
SEM images of PIS05 (**a**), PIS10 (**b**), PIS20 (**e**) and PIS30 (**f**) with KH560 coupling agent, PIS05 (**c**) and PIS10 (**d**) without KH560 coupling agent.

It can be seen from Figure 2 that the composite film prepared by the in situ polymerization method is more conducive to the dispersion of nano-SiO_2_ in the polymer matrix than the film prepared by other methods. With the increase of SiO_2_ content, the interaction between the nanoparticles increases, and the phenomenon of aggregation and even partial agglomeration gradually appears, which causes the particle size of SiO_2_ to increase from about 15 nm in (a) to about 50 nm in (b). Further increasing the amount of SiO_2_ addition, (e) and (f) can see obvious agglomeration. Moreover, the distribution and the size of the particles are not uniform, in, which the largest particle diameter can reach about 400 nm, and the smallest is about 50 nm.

The samples without coupling agents still maintain the advantages of in situ polymerization method of relatively uniform dispersion of inorganic particles. However, compared with the same proportion of SiO_2_ (comparison between a and c), it is not difficult to find that the SiO_2_ nanoparticles of the sample using the coupling agent are relatively small in size, showing a network structure crosslinked with PI and uniformly dispersed in among them. This is because there are three ethoxy groups in the molecular chain of the precursor KH560, all of which are connected to silicon atoms. When the SiO_2_ content is low, the generated SiO_2_ content is also relatively low, and the particle size of SiO_2_ particles is also smaller, gradually forming a continuous network structure of SiO_2_. They penetrate into the PI, and the surface of the SiO_2_ particles is covered with an organic phase, which acts as a physical barrier, making the SiO_2_ nanoparticles difficult to agglomerate. However, when the content of the inorganic phase in the system is too high, as shown in (e) and (f), the use of coupling agents has no significant effect on reducing the size of inorganic nanoparticles. When the content of inorganic matter in the system is too high, SiO_2_ particles increase and the agglomeration effect between SiO_2_ particles is enhanced, so the coupling agent has little effect.

In addition, through comparing the yellow circles in Figure 2b,d, it can be found that the interface between the two phases of the sample with the coupling agent is relatively blurred compared with the sample without the coupling agent. This is because the addition of the coupling agent provides a large number of bonding points for the bond between the organic phase and the inorganic phase, which can improve the compatibility between the organic phase and the inorganic phase, resulting in the relative blurry on the interface between the two phases.

The color of traditional PI films (such as the aromatic PI in the ODA-PMDA system) changes from yellow to brown or even dark brown. Its transmittance in the visible light range (wavelength 400–700 nm) is low, less than 40% at 500 nm, and almost 100% absorption near 400 nm [17]. This is because traditional aromatic PI film has a large number of C-H bonds in the main chain which has strong conjugation and is easy to form intra- and intermolecular charge transfer complexes (CTC). Hence, the transmittance of this film in the visible light region is poor, and the color is darker.

At present, the basic principle for improving the transparency of PI films is to avoid or reduce the existence of conjugated units and reduce the charge transfer effect within or between molecules. The common methods are as follows: The introduction of fluorine-containing substituents into the PI molecular structure inhibits the formation of CTC due to the larger electronegativity of fluorine atoms. The introduction of meta-substitution structure or asymmetric structure into the PI molecular structure can also reduce the formation of CTC. The reduced content of aromatic structure in the PI molecular structure, such as using alicyclic monomers, helps to reduce the probability of CTC formation [18].

We found that the PI synthesized using diamine and dianhydride monomers containing fluorine substituents not only has the best transmission performance among the above three methods but also the cutoff wavelength extends from about 400 nm to about 350 nm. Therefore, TFDB-6FDA system PI is selected as the matrix material to prepare PI/SiO_2_ composite film. The optical transmittance of multiple samples is measured and compared with the traditional aromatic PI (ODA-PMDA system) film.

**Figure 3:**
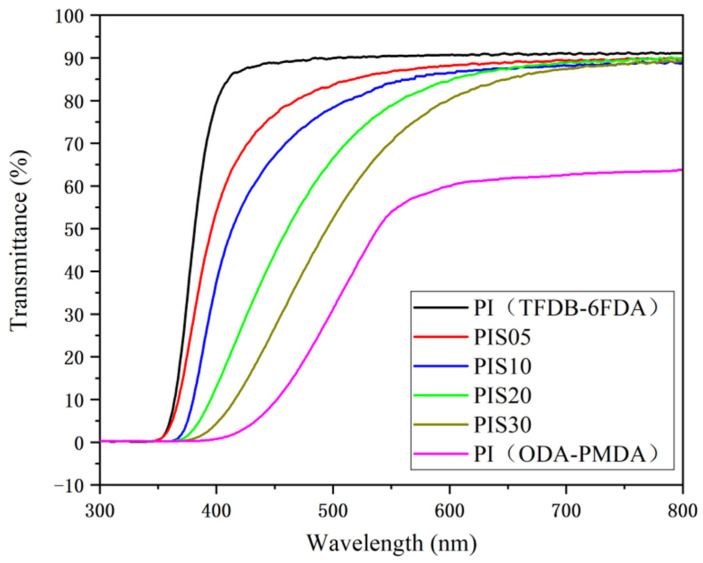
The transmittance of the TFDB-6FDA system PI film, PIS05, PIS10, PIS20, PIS30 and the ODA-PMDA system PI film.

As shown in Figure 3, it can be observed that the transmittance of the TFDB-6FDA system PI film at around 400 nm has reached a high level (above 85%) and can reach 91.4% in the visible light range. However, the transmittance of traditional aromatic PI (ODA-PMDA system) film is almost 0% at about 400 nm and can only reach about 65% in the visible light range. The maximum transmittance of the PI/SiO_2_ composite film with various SiO_2_ doping displays obvious changes in the visible light region. The transmittance of the PIS05, PIS10, PIS20 and PIS30 films are 90.7%, 90%, 88.6%, 86.5%, respectively. However, the transmittances at around 500 nm are 84.7%, 79.8%, 67.4%, 55.6%, respectively, suggesting the doping of SiO_2_ nanoparticles affects the light transmittance of the composite film. With the gradual increase of the SiO_2_ content, an inorganic phase with a large size is gradually formed, which would result in the gradually decreased transmittance of the film. Moreover, the difference is small in the high wavelength region and large in the low wavelength region. This is mainly because as the content of nano-SiO_2_ particles increases, the probability of interaction between nanoparticles is also increasing. The particle size of some particles gradually increases due to agglomeration, and some have exceeded 100 nm, resulting in microphase separation of PI/SiO_2_ composite [19,20], thereby affecting the optical properties of composite materials.

Investigating the mechanical properties of polymer matrix composites, tensile strength, elastic modulus and elongation at break are three essential indicators. As shown in Table 1, as the content of SiO_2_ increases, the tensile strength and elastic modulus of the PI/SiO_2_ films both increase first and then decrease. When the content of SiO_2_ is low, the inorganic particles are well dispersed in the organic polymer matrix. The SiO_2_ nanoparticles and the polymer chain form a crosslinked spatial network structure, which makes the organic and inorganic phases tightly connected, thus having a good mechanical enhancement effect. When the SiO_2_ content reaches 10%, the tensile strength and elastic modulus exhibit the maximum value. However, as the SiO_2_ content continues to increase, on one hand, the increase in the number of inorganic particles makes the distance between the particles gradually decrease. On the other hand, due to the steric hindrance of the polyamic acid molecular chain, longer molecules chain is easy to curl to produce a wrapping effect, which makes the inorganic particles easy to agglomerate and produces larger silica particles. Hence, the distance between the polyimide molecular chains and the gap between the organic and inorganic phases increase at the same time, which reduces the interaction between them, and thus appears the decrease of tensile strength and elastic modulus. With the increase of SiO_2_ content, the elongation at break of the PI/SiO_2_ films gradually decreases due to the rigid SiO_2_ nanoparticles restrict the movement of the PI polymer chain. The agglomeration caused by the increasing number of nanoparticles forms stress concentration points under the action of external forces, which leads to the toughness deterioration of the PI/SiO_2_ films. When the SiO_2_ content reaches 10%, SiO_2_ is still dispersed in the PI matrix in the form of single particles, so the effective contact between the two phases is the largest. The interaction is the strongest, and the effect of enhancing the mechanical properties is the best. Further increasing the content of the inorganic phase, some SiO_2_ nanoparticles will agglomerate, resulting in the reduction of the effective contact between the two phases and weakening of the interaction, thereby reducing the strengthening effect of SiO_2_ on the matrix.

In addition, with the increase of SiO_2_ content, the elongation at break of the composite film gradually decreases, which may be because a large number of SiO_2_ particles destroy the molecular chain structure of polyimide, thus reducing the intermolecular force of polyimide.

**Figure 4:**
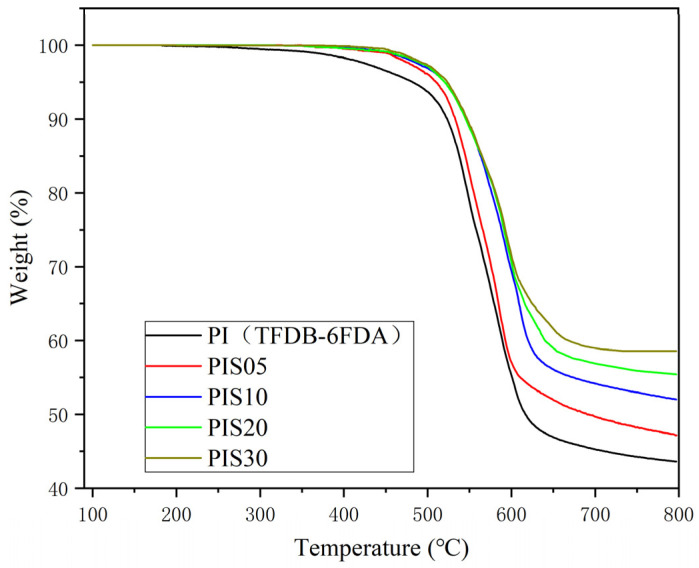
The TGA curves of the TFDB-6FDA system PI film, PIS05 and PIS10.

Figure 4 shows the TGA curves of PI/SiO_2_ composite films with different SiO_2_ content. It can be seen from the figure that with the increase of SiO_2_ content, the decomposition temperature of PI/SiO_2_ material increases, and the thermal decomposition temperature of PI film, PIS5 film and PIS10 film are 517 °C, 522 °C and 525 °C, respectively. In addition, the residual rate at 700 °C also increased with the increase of SiO_2_ content. There are two main reasons for the increase in thermal stability of PI/SiO_2_ composite films: First, SiO_2_ particles have excellent heat resistance. When they are uniformly dispersed in the polyimide matrix at the nanometer level, they will form a crosslinked structure between the carbonyl group in the polyimide molecular chain and the silyl hydroxyl group of SiO_2_. To a certain extent, the thermal vibration of PI is inhibited, thereby helping to improve thermal stability. As the content of SiO_2_ increases, the degree of crosslinking also increases, thermal stability is also improving. Second, the nano SiO_2_ particles are connected to the polyimide main chain through the action of the silane coupling agent, which further restricts the thermal movement of the polyimide macromolecular chain. The more SiO_2_ added, the more the SiO_2_ phase and the PI main chain, the stronger the interaction, which increases the energy required to break the macromolecular chain during the heating process and further improves the thermal stability of the composite film.

As the basic environment for spacecraft to operate in orbit, the space environment has a significant impact on the operation of the spacecraft and various systems. Studies have shown that there are space factors, such as ultraviolet radiation, solar wind protons, vacuum discharge, plasma, micrometeoroids, high and low-temperature cycles, ionizing radiation, and atomic oxygen [21]. The existence of these space factors would have a serious effect on the surface materials, electronic components, surface potential, and operating altitude of the spacecraft. As the covering layer material of spacecraft solar cells, the anti-ultraviolet radiation is the key point to be investigated among the above-mentioned space influence factors.

Solar ultraviolet radiation is the main form of solar electromagnetic radiation [22], and its wavelength range is between 200 and 400 nm. The energy of ultraviolet photons is higher than 376.6 kJ/mol, and the bond energy of organic polymer molecules is generally 250–460 kJ/mol, so the energy of ultraviolet light is sufficient to break the organic chemical bonds, leading to the surface cracking or crosslinking reaction of polymer materials [23] and reducing the mechanical properties of materials. Therefore, this work uses the Cnlight HJ-1402 UV lamp to carry out the simulated space irradiation experiment, continuously irradiating pure PI film and PIS10 composite film for 264 h, the ambient temperature is 21 °C, the humidity is (30 ± 5)%, and the irradiation power is 60 W, The irradiation distance is 20 cm. After the simulated irradiation experiment, the UH4150 ultraviolet-visible-near-infrared photometer and the AGS-X 50 N material universal testing machine were used to investigate the light transmittance and mechanical properties of pure PI and PIS10 samples before and after irradiation.

After simulated ultraviolet irradiation, the maximum transmittance of pure PI decreased from 91.4% before irradiation to 89.8%; the attenuation was about 1.75%. The maximum transmittance of PIS10 decreased from 90% before irradiation to 88.5%; the attenuation was about 1.67%. The transmittance of pure PI and PIS10 both have a small attenuation, but the difference of attenuation is not significant, and the attenuation of PIS10 is slightly lower. This may be because when the doping amount of SiO_2_ is 10%, SiO_2_ still exists in the PI matrix in the form of single particles and does not form a large inorganic phase. Therefore, after UV irradiation, the transmittance and attenuation have little change.

As shown in Table 2, the mechanical properties of pure PI and PIS10 changed greatly after UV irradiation. The attenuation of the tensile strength of PIS10 decreased from 19.76% to 10.65% of pure PI film, the attenuation of elastic modulus decreased from 9.13% to 6.44% of pure PI film, and the attenuation of elongation at break decreased from 13.19% to 8.59% of pure PI film. The introduction of nano-SiO_2_ can effectively resist the effect of ultraviolet irradiation on the composite material. PI is irradiated to produce free radicals. In the process of binding free radicals to protons, chain breakage or crosslinking may occur. When the free radical combines with the proton, the molecular chain breaks from a long-chain to a relatively short fragment, and the decomposition reaction occurs. When two free radicals bind to each other, the two chains become a longer strand fragment, and a crosslinking reaction occurs. The fracture will decrease the tensile strength and elastic modulus, and the elongation at break. However, crosslinking will increase the tensile strength and elastic modulus and decrease the elongation at break. When the elongation at break, tensile strength, and modulus of elasticity decrease at the same time, it indicates that intramolecular fracture occurs, decomposition plays a major role; On the contrary, when the elongation at break decreases, while the tensile strength and modulus of elasticity increase, crosslinking plays a major role. The introduction of nano-SiO_2_ particles into the organic polymer matrix is to improve the bonding energy with the organic polymer [24] by taking advantage of the characteristics of small particle size, large specific surface area, strong hydroxyl reactivity on the surface and excellent anti-irradiation performance of SiO_2_, so as to improve the mechanical properties of the composites.

## 4. Conclusions

Using fluorine-containing diamine TFDB and fluorine-containing dianhydride 6FDA as monomers, a series of PI/SiO_2_ with different SiO_2_ content were synthesized by the in situ polymerization method. The influence of coupling agent and SiO_2_ nanoparticle doping amount on the film structure and performance was studied. It was confirmed that the thermal imidization of the composite film was relatively complete, and the organic/inorganic phase formed a crosslinked network structure. Compared with traditional aromatic polyimide, fluorine-containing polyimide has excellent light transmittance (transmittance can reach 91.4%). The cutoff wavelength of fluorine-containing PI can be extended from about 400 nm to about 350 nm. Doping SiO_2_ nanoparticles into fluorine-containing PI reduces the light transmittance of the composite material as the content of SiO_2_ increases. Doping SiO_2_ nanoparticles into PI film has varying degrees of influence on the mechanical properties, is beneficial to the improvement of the radiation resistance and the thermal performance of PI/SiO_2_ films. In addition, the introduction of a silane coupling agent can effectively inhibit the agglomeration of SiO_2_ nanoparticles, thereby forming a tighter crosslinked structure between organic and inorganic phases. In summary, the fluorine-containing PI/SiO_2_ nanocomposite is a suitable alternative material for flexible substrates in the aerospace field.

## Figures and Tables

**Table 1:** The mechanical properties of PI/SiO_2_ films with various SiO_2_ contents.

Mechanical Properties	Content of SiO_2_ (%)				
	0	5	10	20	30
Tensile strength (MPa)	168	187	198	189	171
Elastic modulus (GPa)	2.08	2.27	2.64	2.19	2.05
Elongation at break (%)	18.2	16.7	12.8	8.9	7.3

**Table 2:** The mechanical properties of the pure PI film and the PIS10 before and after irradiation.

Samples	Tensile Strength (MPa)			Elastic Modulus (GPa)			Elongation at Break (%)		
	Before	After	Attenuation (%)	Before	After	Attenuation (%)	Before	After	Attenuation (%)
PI	168	134.8	19.76	2.08	1.89	9.13	18.2	15.8	13.19
PIS10	198	176.9	10.65	2.64	2.47	6.44	12.8	11.7	8.59

## Supplementary Materials

The following are available online at https://www.mdpi.com/2079-4991/11/3/562/s1, Figure S1: The molecular formula of TFDB, 6FDA and obtained fluorine-containing PI, Figure S2: The XRD spectrum of the SiO_2_ nanoparticles, Table S1: The required amount of PI/SiO2 films with various SiO_2_ contents, Table S_2_: The most commonly used wave numbers of the imide group.
