# Supplementary material for: Synthesis and Properties of Polyimide Silica Nanocomposite Film with High Transparent and Radiation Resistance
Source: Nanomaterials (Basel). 2021 Feb 24;11(3):562. doi: 10.3390/nano11030562 (PMC7995970; doi:10.3390/nano11030562)
Supplement: Supplementary file 1 [file nanomaterials-11-00562-s001.pdf]

Supporting Information

Synthesis and properties of polyimide silica nanocomposite film with high transparent and radiation resistance

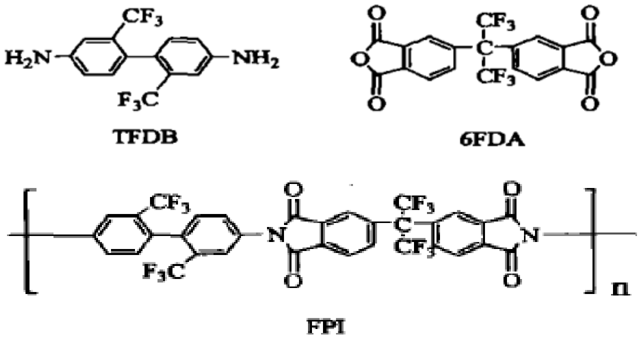

Figure S1. The molecular formula of TFDB, 6FDA and obtained fluorine-containing PI.

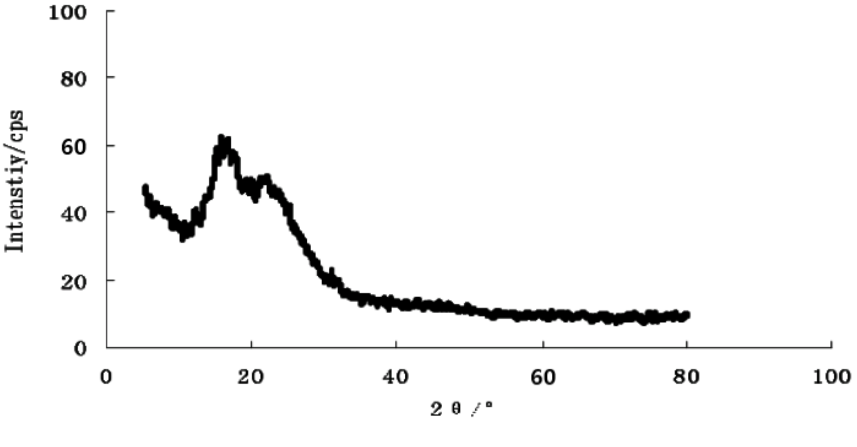

Figure S2. The XRD spectrum of the SiO_2 nanoparticles

Table S1. The required amount of PI/SiO_2 films with various SiO_2 contents

**Samples**	**Mass (g)**					**Percentage of SiO_2 (%)**	**Content of PAA Solution (%)**
	**SiO_2**	**TFDB**	**6FDA**	**DMAc**	**KH560**		
PI	0	3.203	4.532	77.735	0	0	10
PIS05	0.444	3.207	4.529	77.740	0.01332	5.43	9.95
PIS10	0.877	3.210	4.531	77.743	0.02633	10.28	9.96
PIS20	1.937	3.209	4.534	77.738	0.05811	20.01	9.96
PIS30	3.285	3.206	4.530	77.741	0.09855	29.81	9.95

Table S2. The most commonly used wave numbers of the imide group

**Group**	**Characteristic Adsorption Peak (cm^{-1})**	**Type of Adsorption Peak**
Imide	1780	asymmetry extensional vibration of C=O
	1720	symmetrical extensional vibration of C=O
	1380	extensional vibration of C–N
	720	flexural vibration of C=O
